# Intermittent Theta-Burst Stimulation Over the Suprahyoid Muscles Motor Cortex Facilitates Increased Degree Centrality in Healthy Subjects

**DOI:** 10.3389/fnhum.2020.00200

**Published:** 2020-06-16

**Authors:** Guoqin Zhang, Cuihua Gao, Xiuhang Ruan, Yanli Liu, Yuting Li, E Li, Lisheng Jiang, Lingling Liu, Xin Chen, Xinqing Jiang, Guangqing Xu, Yue Lan, Xinhua Wei

**Affiliations:** ^1^Department of Radiology, Guangzhou First People’s Hospital, Guangzhou Medical University, Guangzhou, China; ^2^The Second Affiliated Hospital, South China University of Technology, Guangzhou, China; ^3^Department of Rehabilitation Medicine, Guangzhou First People’s Hospital, Guangzhou Medical University, Guangzhou, China; ^4^Department of Rehabilitation Medicine, Beijing Tiantan Hospital, The Capital Medical University, China National Clinical Research Center for Neurological Diseases, Beijing, China

**Keywords:** theta-burst stimulation, repetitive transcranial magnetic stimulation, swallowing, degree centrality, suprahyoid muscles

## Abstract

Theta-burst stimulation (TBS), a variant of repetitive transcranial magnetic stimulation (rTMS), can potentially benefit the treatment of swallowing disorders. However, the after-effects of TBS on the swallowing motor cortex remain uncertain. The newly developed graph-based analysis of the centrality approach has been increasingly used to explore brain networks. The purpose of this study was to identify degree centrality (DC) alterations in the brain network after different TBS protocols were performed over the suprahyoid muscles motor cortex in healthy subjects. A total of 40 right-handed healthy subjects (mean age: 23.73 ± 2.57 years, range: 21–30, 20 females) were included in this study and randomly assigned to two groups, including the continuous TBS (cTBS) group and the intermittent TBS (iTBS) group. All of the subjects underwent resting-state functional magnetic resonance imaging (rs-fMRI) scanning before and after TBS implementation. Compared to the baseline, cTBS resulted in increased DC values in the left inferior frontal gyrus (*P* < 0.01). In the iTBS group, decreased DC was observed in the left cerebellum and left medial frontal gyrus; However, increased DC was observed in several brain areas including the right superior temporal gyrus, right superior frontal gyrus, right postcentral gyri and left paracentral lobule (*P* < 0.01). These results indicated that cTBS mainly results in increasing DC in the ipsilateral. However, iTBS is capable of facilitating the excitability of the swallowing motor cortex and increasing the connectivity of multiple brain regions, including the bilateral sensorimotor network, and might have therapeutic potential in the treatment of swallowing disorders.

## Introduction

Swallowing is one of the most complex sensorimotor tasks involving a widely distributed neuronal network, including different levels of the central nervous system from the bilateral cerebral cortex to the medulla oblongata and many of the cranial nerves (Kern et al., 2001; Martin et al., 2001; Ertekin and Aydogdu, 2003; Cicala et al., 2019). A swallowing disorder (i.e., dysphagia) may occur as a result of disruption at any one of these levels (Rangarathnam et al., 2014). Many diseases or disorders can lead to dysphagia (Doeltgen et al., 2015), which is a major risk factor for pneumonia after stroke and has also been associated with a prolonged hospital stay, high mortality, and other terrible outcomes (Langdon et al., 2007). How to effectively treating dysphagia is a challenge in clinical practice. Many traditional treatments have been used in clinical to recover the swallowing function of patients with dysphagia; however, the evidence of their effectiveness is limited or controversial (Ashford et al., 2009; Geeganage et al., 2012).

Repetitive transcranial magnetic stimulation (rTMS) is a noninvasive neurostimulation technique that is based on the law of electromagnetic induction for delivering electric field pulses into the brain and has been increasingly used to investigate the neural mechanisms of swallowing and dysphagia (Khedr et al., 2009; Vasant et al., 2014; Pisegna et al., 2016). Transcranial magnetic stimulation (TMS) can lead to short- or long-term aftereffects that facilitate or impede neuronal excitability, depending on the site of stimulation, anatomy, and stimulation parameters (Diana et al., 2017). High-frequency rTMS (5–25 Hz) has typically shown excitatory effects, whereas low-frequency rTMS (~1 Hz) usually reduced excitability (Pascual-Leone et al., 1994; Chen et al., 1997), reminiscent of long-term potentiation (LTP) and long-term depression (LTD), respectively. More recently, an exciting novel form of rTMS known as theta-burst stimulation (TBS) employs lower-intensity stimuli, a more brief stimulus time and a smaller number of pulses to regulate motor cortical excitability (Huang et al., 2005). Different patterns of TBS delivery, including continuous TBS (cTBS) and intermittent TBS (iTBS), produced opposite effects on the synaptic efficiency of the stimulated cortex (Huang et al., 2005). A prolonged LTP-like increase in motor cortical excitability was generated by iTBS, whereas the cTBS produced the effect of a prolonged LTD-like decrease in excitability (Gamboa et al., 2010; Di Lazzaro et al., 2011). Moreover, several studies have suggested that TBS is a more effective method than traditional rTMS in improving cerebral function, ability to function, and neurological deficits in stroke patients (Ackerley et al., 2010; Hsu et al., 2012). Also, one other study had confirmed that TBS can improve the motor function obstacle with a sequence of iTBS stimulation on only the affected hemisphere motor cortex (M1; Suppa et al., 2008). However, alterations in the aftereffects across the bilateral hemispheres and the connectivity of brain regions following the application of different patterns of TBS remain unclear.

The interhemispheric competition model has been broadly accepted in motor function recovery. Based on the theory, the reasons for motor deficits in stroke patients include decreased output from the affected hemisphere and excess interhemispheric inhibition from the unaffected hemisphere to the affected hemisphere (Takeuchi and Izumi, 2012). Therefore, a strategy for improving motor deficits can be achieved by increasing the excitability of the affected hemisphere or inhibiting the excitability of the unaffected hemisphere. However, in contrast to most somatic functions, the swallowing muscles are innervated by the bilateral motor cortices (Mistry et al., 2007). Studies have indicated that high-frequency rTMS applied to the affected hemisphere can directly improve cortical excitability (Kim et al., 2004), and there was no significant alteration in excitability of the ipsilateral or contralateral pharyngeal motor cortex after cTBS implementation (Mistry et al., 2007). Therefore, the neural mechanisms of TBS in the swallowing motor cortex remain incompletely understood.

In recent years, resting-state fMRI (rs-fMRI) has been widely used in exploring alterations in brain spontaneous neuronal activity (Wiebking et al., 2011; Wee et al., 2014). In particular, degree centrality (DC), a graph-based measurement of whole-brain functional connections, has received substantial attention because it considers the relationship of a given region with the entire functional connectome at the voxel level (Zhong et al., 2019). It has been proposed that the DC was the most dependable metric among several nodal network metrics (Buckner et al., 2009; Wang et al., 2011). In the DC analysis, the greater the number of connections a region has, the more powerful the connections of this region are (van den Heuvel and Hulshoff Pol, 2010). Therefore, when the nodes or DC values change in a region, it indicates that there are abnormal connections for the functional synchronization with other brain regions (Stam et al., 2009). The DC method has been applied in mental and neurological disorders, such as Parkinson’s disease and epilepsy (Wang et al., 2017; Zhong et al., 2019). To our knowledge, no study to date has investigated DC alterations induced by TBS application to the swallowing cortex in subjects.

The suprahyoid muscles are reliable target muscles for swallowing outcome measurements and treating dysphagia (Wichniak et al., 2011; Kothari et al., 2017). The primary aim of the present study was to explore the aftereffects induced by different patterns of TBS over the cortex of the suprahyoid muscles by using a centrality analysis at the whole-brain level in healthy subjects. Several studies have reported that iTBS and cTBS produced opposite effects on the synaptic efficiency in the stimulated cortex (Huang et al., 2005; Suppa et al., 2008; Verin et al., 2012; Wu et al., 2012). Based on these results, we hypothesized that different patterns of TBS will induce opposite patterns of spontaneous neuron activity, and we presume that the DC values may be used as a biomarker to identify the underlying neural basis for TBS.

## Materials and Methods

### Participants

Forty young healthy subjects (mean age: 23.73 ± 2.57 years, range: 21–30, 20 females) from the local colleges were recruited through advertisements and randomly assigned to two groups: the cTBS group and the iTBS group (see Table 1). All participants were right-handed as assessed by the Edinburgh handedness inventory (Oldfield, 1971). None of the subjects had a history of neurological and/or psychiatric disorders, swallowing disorders or ear, nose, or throat surgery. No subjects were using medications that may influence the neuromuscular system. These participants did not regularly smoke or drink tea. The other exclusion criteria were: (1) being unable to complete the experiments; and (2) having a contraindication for the MRI examination and TBS treatment. The participants gave written informed consent for the study, which was approved by the local Medical Ethics Committee of Guangzhou First People’s Hospital and performed following the ethical guidelines of the Declaration of Helsinki.

**Table 1:** Demographics of the participants.

Variables	cTBS group (*n* = 20)	iTBS group (*n* = 20)	*p*
Gender (Male/Female)	10/10	10/10	1.00^a^
Age (Mean ± SD Years)	23.60 ± 2.23	22.95 ± 2.67	0.60^b^

*cTBS, continuous theta-burst stimulation; iTBS, intermittent theta-burst stimulation; SD, standard deviation. ^a^*p*-value obtained by using the Chi-Square test between-group cTBS and group iTBS participants. ^b^*p*-value for comparison between group cTBS and group iTBS participants obtained by using an independent-sample t-test*.

### Transcranial Magnetic Stimulation and Electromyographic Measurements

The detailed process of TMS has been described in previous studies (Lin et al., 2017; Ruan et al., 2017). Briefly, subjects were comfortably seated. A pair of electrodes (Yiruide, Wuhan, China) was placed on the surface of the first dorsal interosseous (FDI) muscle on the right hand to record motor evoked potentials (MEPs). Electromyography (EMG) data were recorded from the suprahyoid muscle surface *via* surface electrodes at the same time. All electrodes were connected to an EMG recording system (Yiruide, Wuhan, China) with a preamplifier and amplifier.

The TBS pulses were performed using a hand-held figure-of-eight coil (outer diameter, 70 mm) connected to a magnetic super rapid stimulator (Yiruide CCY-IA, Wuhan, China) over the regions of interest on the scalp. The optimal locations for cortical stimulation were guided by a stereotactic online navigation system (Softaxic Optic, Canada, NDI). The stimulating coil was placed on one side of the scalp and held tangentially to the scalp surface with the coil handle pointing backward and laterally 45° away from the anterior-posterior axis. Then, the monopulse stimulation was initially triggered at 60% maximum output intensity and gradually increased until the visible abduction of the thumb was observed. This stimulation intensity was maintained and the coil was gradually moved with a spacing of 0.5–1.0 cm. When five consecutive stimuli evoked the largest amplitude of the MEP in the same cortex, this was considered the “hot spot.” After the location of the hot spot was determined, the stimulation intensity was gradually decreased until the MEP response was ≥200 μV in the abductor pollicis brevis muscle following at least five of 10 consecutive stimulations, with the coil position being maintained invariant during the process. This stimulation intensity was verified as the active motor threshold (AMT) by the navigation system. Then, 70% of the AMT was delivered, and the coil was moved around an area 0.5–1.0 cm in the anterior and lateral directions. The location of the maximum MEP after five consecutive stimulations was regarded as the optimal point for the cortical mapping of the suprahyoid muscle motor cortex in each hemisphere. The navigation system recorded the optimal point to generate a uniform position of stimulation in the subsequent studies.

TBS was performed using the cTBS pattern described by a previous study (Huang et al., 2005). In the current study, each TBS protocol consisted of three stimulation pulses delivered at 50 Hz and repeated every 200 ms (5 Hz). In cTBS, a 40-s train of cTBS was administered (600 pulses). In the iTBS paradigm, 20 trains of two bursts were administered at 10-s intervals for approximately 190 s (600 pulses in total). The stimulation intensity was set at 80% of the AMT for the FDI.

Our study included the following experiments: experimental protocol A: cTBS with the stimulation site at the motor cortex of the suprahyoid muscles in the left hemisphere; experimental protocol B: iTBS with the stimulation site at the same location as the cTBS protocol.

### MR Data Acquisition

The fMRI measurements were obtained on a 3-Tesla Siemens Verio scanner (Siemens, Erlangen, Germany) with a 16-channel phased-array head coil. The baseline MRI scan was performed 2 h before the TBS implementation, and the post-TBS scan was completed within 30 min after TBS. The rs-fMRI parameters were as follows: repetition time (TR) = 2,000 ms, echo time (TE) = 21 ms, flip angle (FA) = 90°, field of view (FOV) = 240 mm × 240 mm, matrix = 64 × 64, slice thickness = 4.0 mm, 33 axial slices, and voxel size = 3.75 mm ×3 0.75 mm × 4.0 mm.

Structural images were acquired using a magnetization-prepared rapid gradient echo (MPRAGE) sequence with the following parameters: TR = 2,530 ms, TE = 2.93 ms, FA = 7°, FOV = 256 mm × 256 mm, and a slice thickness of 1.0 mm with no gap.

During the period of rs-fMRI scanning, participants were asked to relax with their eyes closed but not to fall asleep and asked to empty their minds of anything in particular. After MR scanning, they completed a simple questionnaire to confirm their wakefulness during the scanning.

### MR Data Preprocessing

The resting-state fMRI data preprocessing was carried out using the Data Processing Assistant for rs-fMRI (DPARSF^1^; Yan and Zang, 2010). Most of the functions are based on Statistical Parametric Mapping software (SPM8^2^). Consistent with our previous work (Ruan et al., 2017), for fMRI signal equilibrium and for the subjects to adapt to the scanning noise, the first 10 functional volume images of each subject were removed. The slice timing correction was conducted for the remaining 190 volumes of images. All subjects were allowed head motion only within the scope of 2 mm of movement or 2° rotation in any direction. Then, the 3D structural images were placed into the standard Montreal Neurological Institute (MNI) space provided by SPM8, which was used for spatial normalization with a resampling voxel size of 3 mm × 3 mm × 3 mm. The white matter, cerebral spinal fluid signal, and the Friston 24 head-motion parameters were regressed out from the time series of every voxel. Then, a Gaussian kernel of 4-mm full-width at half-maximum (FWHM) was used in spatial smoothing, the effects of low-frequency drift and high-frequency noise (e.g., respiratory and cardiac noise) were reduced by temporal bandpass filtering (0.01–0.08 Hz) of the functional data, and the linear trend of the data was removed (Biswal et al., 1995).

### DC Calculation

The Resting-State fMRI Data Analysis Toolkit (REST) V1.8 (Zuo et al., 2012) was used for the calculation of voxelwise DC by counting the number of functional connections. First, all pairs of brain voxels in the defined mask were calculated by Pearson’s correlation coefficients (r). Then, we obtained an n × n matrix of Pearson correlation coefficients depicting the whole-brain functional connectivity pattern, where n is the voxel number of the whole-brain mask. Furthermore, Pearson’s correlation data were transformed into normally distributed Fisher’s Z-scores. The sum of Z-scores, representing the weight of the DC values, was computed between a given brain voxel and all other voxels. To obtain each participant’s graph, we constructed the whole-brain functional network by choosing a threshold of 0.25 at *P* ≤ 0.001 to eliminate possible spurious connectivity as previously described (Li et al., 2016). The standardized weighted DC maps were acquired by subtracting the mean value and then dividing by the standard deviation within the whole gray matter mask. Finally, before analyzing the DC, the resulting data were spatially smoothed with a 6-mm FWHM Gaussian kernel.

In the present study, DC maps were calculated based on two additional correlation thresholds (i.e., 0.2 and 0.3) to examine whether our primary results were dependent on the chosen threshold because the choice of the threshold was arbitrary (Buckner et al., 2009).

### Statistical Analysis

The participants’ age and sex were compared between the iTBS and cTBS groups using the Wilcoxon-Mann–Whitney test; *P* < 0.05 was considered to be statistically significant. A paired *t*-test was performed to investigate the difference between the post-TBS and baseline conditions in each group; *P* < 0.01 was considered to be statistically significant. All results were presented at the statistical threshold of *P* < 0.01 using the AFNI AlphaSim program correction, as determined by Monte Carlo simulations^3^, which was used to calculate the probability of false-positive detection while accounting for both the individual voxel probability thresholding and cluster size (FWHM = 6 mm). Using this program, clusters of greater than 40 voxels were applied to the resulting statistical map at a corrected significance level of *P* < 0.01 (Wang et al., 2012). Finally, we compared the DC values extracted from the brain areas with significant differences in the DC maps for the two groups.

## Results

### Demographics Comparison

No significant differences were noted between the cTBS and iTBS groups regarding sex and age (Table 1).

### Alterations in DC Between Baseline and Post-cTBS

**Figure 1:**
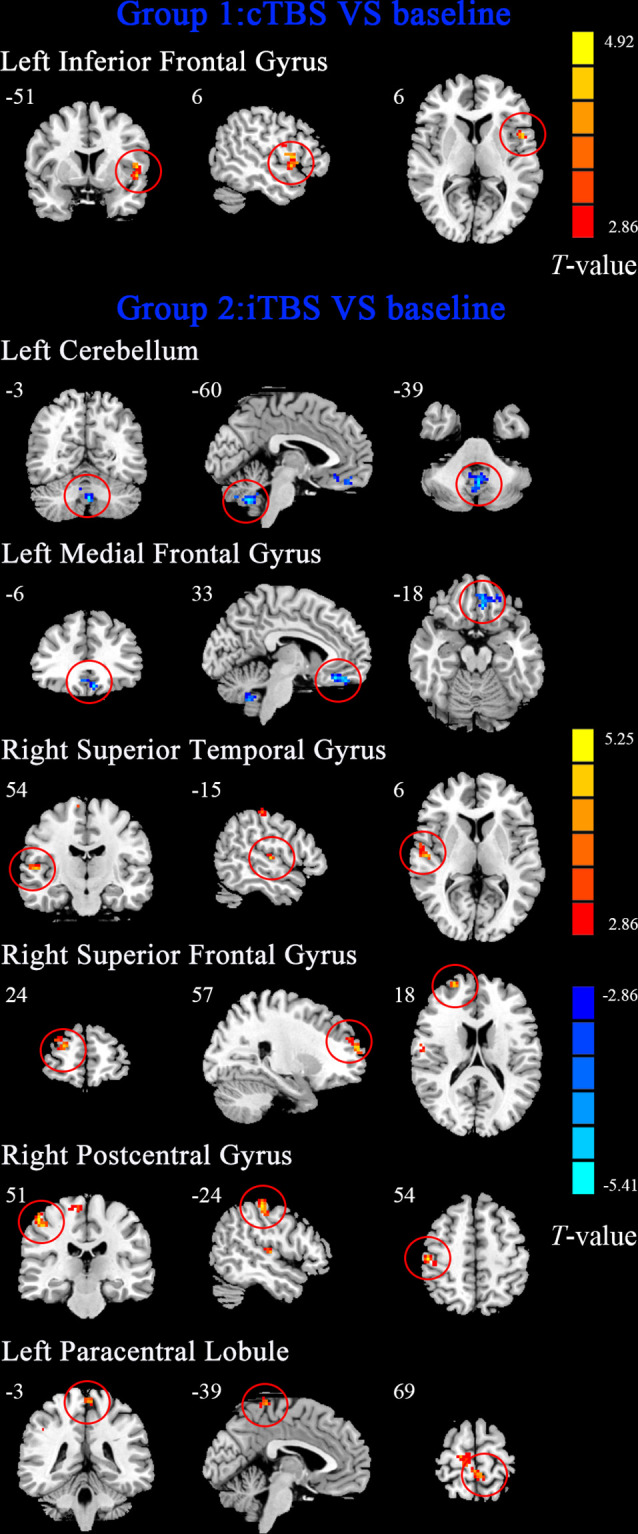
The maps showed that the alteration of degree centrality (DC) after continuous theta-burst stimulation (cTBS) and intermittent theta-burst stimulation (iTBS) protocols on the motor cortex of suprahyoid motor cortex in healthy subjects. Regions with red color represent significantly increased DC values in the theta-burst stimulation (TBS) compared with the baseline, and blue indicates the opposite (*p* < 0.01, corrected). The details are described in Table 2. The color bar indicates the *t*-score.

Compared with baseline, after cTBS was applied to the left suprahyoid motor cortex, we observed increased DC values in the left inferior frontal gyrus (BA 48; *P* < 0.01; Figure 1, Table 2).

**Table 2:** Brain regions with alteration of degree centrality after cTBS and iTBS protocols on the motor cortex of suprahyoid motor cortex.

Brain region	BA	Cluster size (voxels)	Peak MNI coordinates (mm)			*T*-value
			*X*	*Y*	*Z*	
**Post-cTBS vs. baseline**
The Left Inferior Frontal Gyrus	48	139	−51	6	6	4.4728
**Post-iTBS vs. baseline**
The Left Cerebellum	N/A	73	−3	−60	−39	−4.9962
The Left Medial Frontal Gyrus	11	67	−6	33	−18	−5.4116
The Right Superior Temporal Gyrus	48	41	54	−15	6	4.7811
The Right Superior Frontal Gyrus	10	77	24	57	18	4.7482
The Right Postcentral_Gyrus	3	65	51	−24	54	5.1766
The Left Paracentral_Lobule	4	75	−3	−39	69	4.3511

*cTBS, continuous theta-burst stimulation; iTBS, intermittent theta-burst stimulation; BA, Brodman’s area; MNI, Montreal Neurological Institute; N/A, not available*.

### Alterations in DC Between Baseline and Post-iTBS

The results are shown in Figure 1 and Table 2. Compared to baseline, application of iTBS to the left suprahyoid motor cortex resulted in decreased DC in the left cerebellum and left medial frontal gyrus (BA 11), and increased DC were seen in the right superior temporal gyrus (BA 48), right superior frontal gyrus (BA 10), right postcentral gyrus (BA 3), and left paracentral lobule (BA 4; *P* < 0.01).

### DC Comparison Across the Different Thresholds

No significant differences in the distribution of brain regions and voxel sizes were obtained when the calculation threshold was set at 0.2, 0.25, and 0.3 (*P* < 0.05; see Supplementary Figure S1 in the online supplement).

## Discussion

TBS has been proven to be an effective protocol for modulating the excitability of the motor cortex. Few reports have explored the aftereffects of TBS over the swallowing cortex using MEP measurements (Lin et al., 2017). In the current study, we first measured the DC values at the whole-brain level to investigate the aftereffect of different patterns of TBS over the suprahyoid muscle motor cortex. The main findings were that, compared with the baseline: (a) cTBS led to increased connectivity in the left inferior frontal gyrus; and (b) iTBS mainly induced increased DC in multiple brain regions, including the bilateral somatosensory cortices. These observations are likely to have a neurophysiological basis for the application of TBS in the treatment of swallowing deficits.

### Alterations in DC Between Baseline and cTBS

It is well known that cTBS is a suppressive stimulus pattern that can rapidly reduce the excitability of the motor cortex and this effect is sustained for 30 min (Huang et al., 2007). However, similar to another study (Mistry et al., 2007), we did not find alterations in DC in the left primary sensorimotor cortex where the cTBS was delivered. The suppressive effect of cTBS has been related to particular patterns and intensities (Mistry et al., 2007). Another explanation is that we did not determine the dominant cortex before we performed the cTBS, which might have influenced the physiologically measurable effects in the present results. Interestingly, we observed increased DC in left inferior frontal gyrus (BA 48), which belongs to a module of the swallowing network that includes the inferior frontal gyrus, S2, corpus callosum, basal ganglia and thalamus (IFG-S2-CC-BGTHAL module), plays a role in sensory cues within motor sequences (Mosier and Bereznaya, 2001), and has a positive correlation with another module consisting of primary sensorimotor areas and the cingulate gyrus (Mosier and Bereznaya, 2001). Hence, we speculate that increased DC in the left inferior frontal gyrus means that there was a compensatory activation that occurred after the cTBS over the left swallowing motor cortex. Based on the above results, we speculated that the cTBS had no significant aftereffects on the stimulated swallowing motor cortex and the contralateral homologous area but mainly led to the increased functional connectivity in the part of the IFG-S2-CC-BGTHAL module.

### Alterations in DC Between Baseline and iTBS

As expected, we observed increased DC in the swallowing somatosensory cortex, including the right postcentral cortex and left paracentral lobule. A body of research has indicated that the primary somatosensory cortex plays a role in the process of the initiation and control of swallowing (Plant, 1998; Hamdy et al., 1999; Kern et al., 2001; Ertekin and Aydogdu, 2003). Notably, in the present study, even though the iTBS was performed in the left hemisphere, the functional connectivity of the bilateral somatosensory cortex cortices was increased, which was contradictory to the traditional theory of the motor cortex competitive inhibition (Takeuchi and Izumi, 2012). This discrepancy might be explained by suggesting that the swallowing was dominated by the bilateral somatosensory cortices (Suppa et al., 2008). Furthermore, we noticed that many brain areas demonstrated increased DC, including the right superior middle temporal gyrus and right superior frontal gyrus after iTBS implementation. We suggest that iTBS facilitated increases in functional connectivity of the brain cortex. Another study also indicated that the neural network of the bilateral deglutition cortices was more easily facilitated than inhibited (Singh et al., 2009). Also, the DC of the cerebellum was decreased in the present study. The cerebellum has a bidirectional connection with the lower brainstem and cerebrum and regulates the accuracy and coordination of swallowing (Hamdy et al., 2000). However, its role in human swallowing was until now unclear (Hamdy et al., 1999). Based on the above findings, we suggest that iTBS facilitated bilateral increases in functional connectivity in multiple brain areas, and these aftereffects might be meaningful for the selection of TBS treatment for swallowing disorders.

There are several limitations to the present study. First, we did not include a sham stimulation group in this study. Second, to balance the quality of the imaging data and the cooperation of participants, we did not prolong the scanning time until the aftereffect of the TBS dissipated vanished. Third, the sample size was relatively small, which might have influenced the reliability of the results. Finally, static analysis for the rs-fMRI data was used in this study; however, dynamic functional connectivity potentially reflects variations that more accurately represent the dynamic nature of the brain (Boissoneault et al., 2018).

In summary, this study combined rs-fMRI with TBS to identify the aftereffects of TBS on spontaneous neuronal activity in healthy subjects. The cTBS mainly reduced the functional connections in the ipsilateral auxiliary motor areas and decreased DC in the contralateral sensorimotor area and sensory pathway at the same time but increased the ipsilateral cognitive attention network functional connectivity. The iTBS facilitated an increase in the centrality of brain networks, especially in the bilateral sensorimotor cortices. The present results suggest that iTBS may be used as a tool to increase brain functional connectivity in the bilateral hemispheres. Our results provide novel insights into the neural mechanisms of different TBS protocols on the swallowing cortex and suggest possible ways of treating swallowing problems.

## Data Availability Statement

All datasets generated for this study are included in the article/Supplementary Material.

## Ethics Statement

The studies involving human participants were reviewed and approved by the local Medical Ethics Committee of Guangzhou First People’s Hospital and performed in accordance with the ethical guidelines of the Declaration of Helsinki. The patients/participants provided their written informed consent to participate in this study.

## Author Contributions

XW contributed to the experimental design and writing of the manuscript. GZ, CG, and XR were involved in the literature review, data collection, and writing of the manuscript. YLi, LL, and XC contributed to the analysis of MRI data. LJ, EL, and YLiu were involved in the data collection. GX, XJ, and YLa contributed to the experimental design and the writing process.

## Conflict of Interest

The authors declare that the research was conducted in the absence of any commercial or financial relationships that could be construed as a potential conflict of interest.

## Footnotes

^1^http://rfmri.org/dpabi

^2^http://www.fil.ion.ucl.ac.uk/spm

^3^http://afni.nimh.nih.gov/pub/dist/doc/manual/AlphaSim.pdf

## Supplementary Material

FIGURE S1 No significant differences in the distribution of brain regions and voxel sizes were obtained when the calculation threshold was set at 0.2, 0.25, and 0.3 (*P* < 0.05) between cTBS (**S1A**) and iTBS (**S1B**).
