# Supplementary figures and images for: Intermittent Theta-Burst Stimulation Over the Suprahyoid Muscles Motor Cortex Facilitates Increased Degree Centrality in Healthy Subjects
Source: Front Hum Neurosci. 2020 Jun 16;14:200. doi: 10.3389/fnhum.2020.00200 (PMC7309184; doi:10.3389/fnhum.2020.00200)

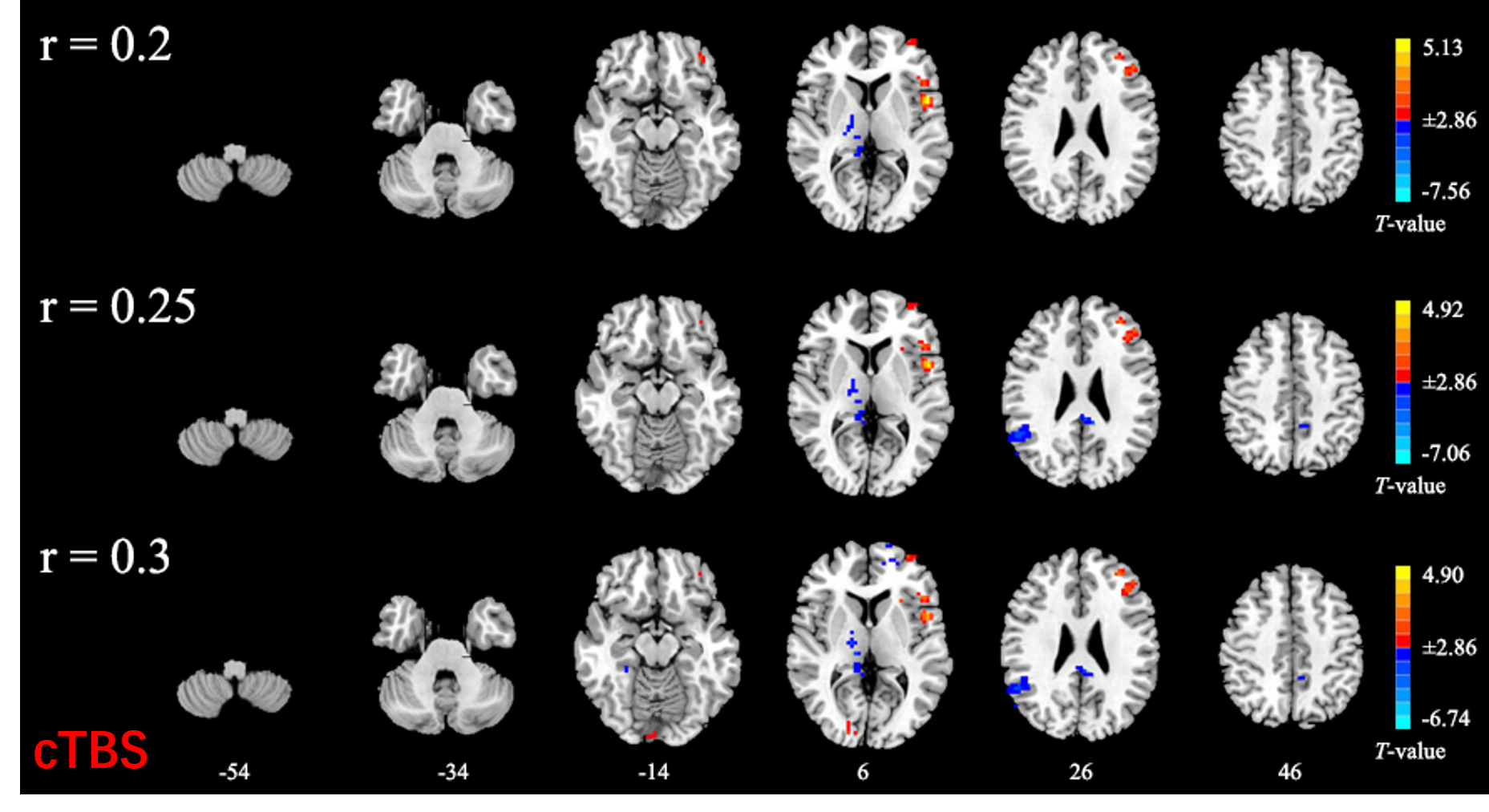

Supplement: FIGURE S1 — No significant differences in the distribution of brain regions and voxel sizes were obtained when the calculation threshold was set at 0.2, 0.25, and 0.3 (P < 0.05) between cTBS (S1A) and iTBS (S1B). [file Image_1.tif]

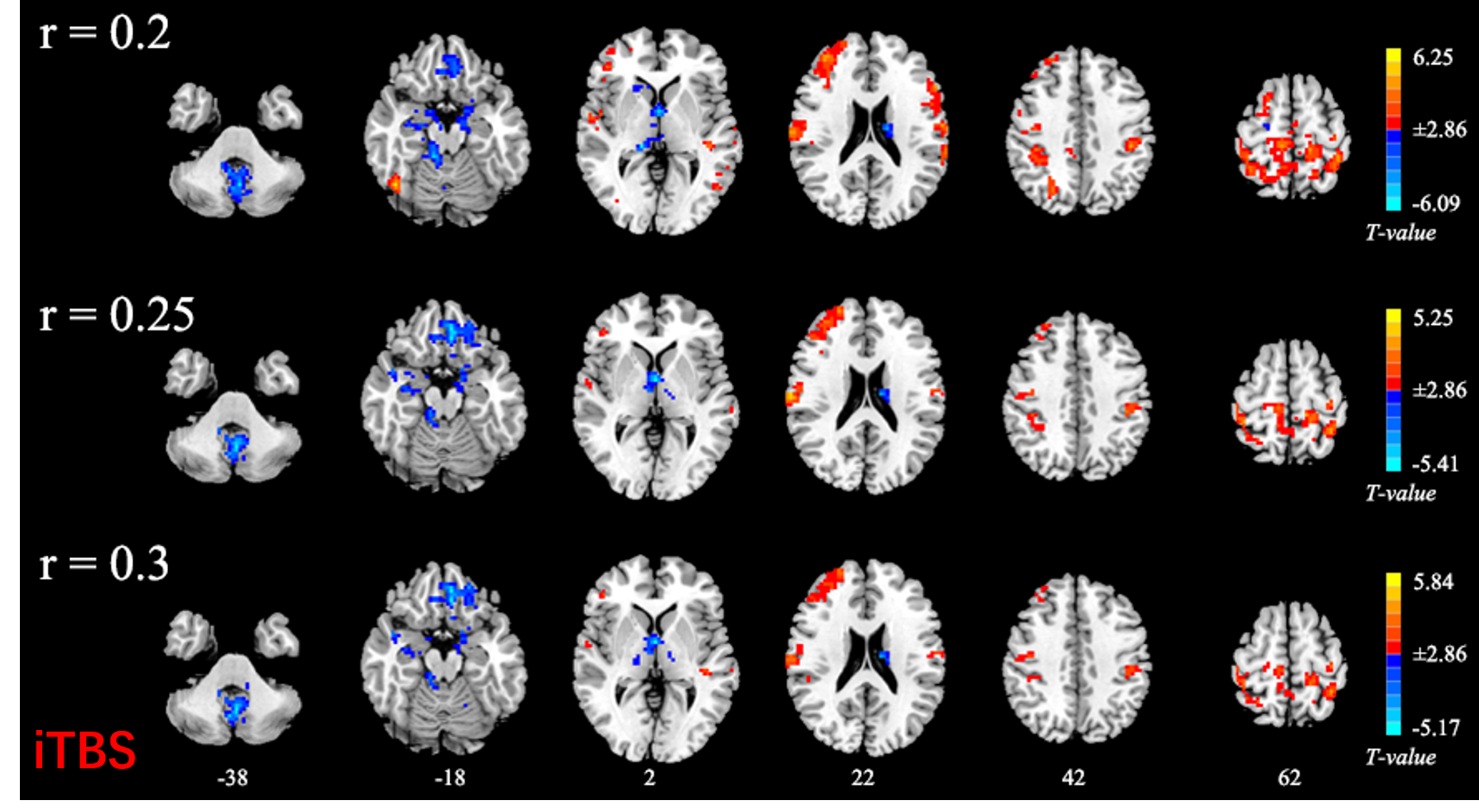

Supplement: Supplementary file 2 [file Image_2.tif]
